# Continuation or Withdrawal of Life-sustaining Therapies After Acute Anoxic Events in Childhood and Adolescence: Can Early MRI Predict Particularly Severe Outcomes?

**DOI:** 10.1007/s00062-026-01657-1

**Published:** 2026-05-07

**Authors:** Katharina Staudt, Martin Staudt, Irina Mader, Melanie Hessenauer, Ingeborg Kraegeloh-Mann, Samuel Groeschel

**Affiliations:** 1 Children’s Hospital Traunstein, Traunstein, Germany; 2 Dept. Pediatric Neurology, University Children’s Hospital Tübingen, Tübingen, Germany; 3 Center for Pediatric Palliative Care, Dr von Hauner Children’s Hospital, Ludwig-Maximilians-Universität München, Munich, Germany; 4 Dept. Pediatric Radiology, University Children’s Hospital Basel, Basel, Switzerland; 5 Center for Pediatric Neurology, Neurorehabilitation and Epileptology, Schön Clinic Vogtareuth, Vogtareuth, Germany

**Keywords:** Anoxia, Hypoxia, Prognosis, Brain imaging, Diffusion MRI

## Abstract

**Purpose:**

Withholding or withdrawing life-sustaining therapies is often discussed when children or adolescents suffer severe anoxic events. Previous studies have demonstrated that early MRI can identify patients with major neurological sequelae, but not whether, within a cohort with major neurological sequelae, early MRI can predict particularly severe outcomes. This was the purpose of the present study.

**Methods:**

We retrospectively assessed MRI (analyzing 14 brain regions for signal abnormalities on diffusion, T1, T2 and FLAIR) of 38 patients who had suffered acute anoxic events between 8 months and 17 years of age and had required inpatient neurorehabilitation, looking for differences between patients with “particularly severe outcomes” (the 22/38 patients who were still unable to interact with their environment 22 weeks after the event) and patients with “less severe outcomes” (the 16/38 patients who had regained this ability within 22 weeks).

**Results:**

Prediction of “particularly severe outcome” was optimal using diffusion MRI obtained on days 4–5 after the event (available in 11 patients, 7 with particularly severe outcomes), when all patients with diffusion restrictions in any of the following regions—putamen (4/7), caudate nucleus (4/7), globus pallidus (6/7), or substantia nigra (4/7)—later showed particularly severe outcomes. In contrast, identification of “less severe outcome” was optimal using MRI obtained between days 6–9 after the event, when absence of diffusion restrictions in the cerebral white matter always predicted “less severe outcome”.

**Conclusion:**

Early MRI can identify patients with particularly severe outcomes—and thus can contribute to discussions about the continuation or withdrawal of life-sustaining therapies in the acute phase after anoxic events.

## Introduction

Anoxic brain injuries in childhood and adolescence, like near-drowning accidents, can have devastating neurological consequences, leading to life-long disabilities [1]. Therefore, in particularly severe cases, withholding or withdrawing life-sustaining therapies can be considered, especially during the first days after the event when the patients are still dependent on intensive care technologies such as mechanical ventilation.

Magnetic resonance imaging (MRI) has the potential to predict neurological outcome during this critical period. This has been shown for neonatal asphyxia [2, 3] and for anoxic events occurring during childhood and adolescence [4–12]. In these studies, “poor” or “unfavorable” outcome was classified as “with significant disability”, e.g. as cerebral palsy with a Gross Motor Function Classification System Score > 1 [3] or > 2 [2], as IQ < 85 [2], as Glasgow Outcome Scale of > 3 [6] or as Pediatric Cerebral Performance Category of > 2 [9, 12] or > 3 [5, 7, 10, 13]. These approaches are quite helpful for the early identification of patients who will require neurorehabilitation or long-term specialized care. They might, however, not contribute to discussions whether life-sustaining therapies should be withheld or withdrawn while the patient is still dependent on intensive care technologies, since these discussions only take place in the most severely affected patients. Whether MRI can also provide this more specific information has, to our knowledge, never been addressed in previous studies.

Therefore, in this study, as a first step to close this gap, we asked whether, within a cohort of children and adolescents severely affected by an anoxic event (i.e., requiring inpatient neurorehabilitation following the event), an MRI early after the anoxic event could discriminate between patients with *particularly severe outcomes* and those with *less severe outcomes*.

## Patients and Methods

**Fig. 1:**
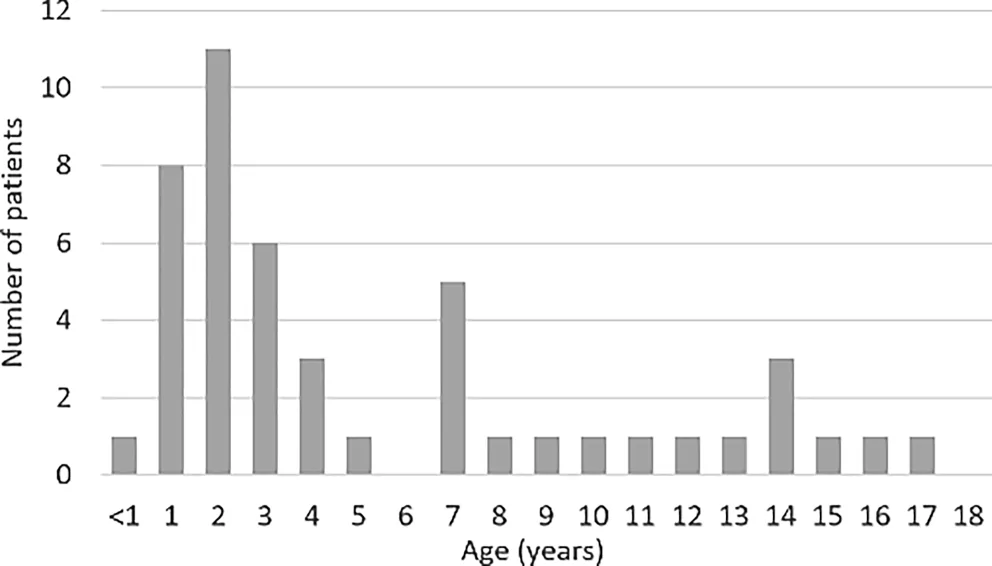
Age distribution. Gray bars indicate the number of patients included for each year of age (at the time of the anoxic event)

In this retrospective analysis, we included all 47 children and adolescents (15 female) who underwent inpatient neurorehabilitation at the Schön Clinic Vogtareuth, a large specialized center for pediatric neurorehabilitation, between February 2005 and September 2019 following acute anoxic events. The ages at the time of the anoxic event (Fig. 1) ranged from 8 months to 17 years (median 3.65 years). The main causes for the anoxia were near-drowning accidents (18/47), foreign-body aspirations (8/47), and cardiac arrests due to (suspected) cardiac arrhythmias (8/47). Patients with pre-existing diagnoses implying a possible influence on brain structure or development (e.g., chromosomal aberrations, or congenital heart disease) or with additional trauma (e.g. shaken baby syndrome) were excluded. In order to assess major neurological sequelae, we reviewed the rehabilitation hospital charts for the presence of dystonia/spasticity, epilepsy, tracheostomies and gastrostomies, and used Fisher exact tests for searching for differences between outcome groups (see below), with a cut-off of *p* < 0.05 indicating significance.

Neurological outcomes were measured using RemiPro (“Remission Profile”) [1, 14], a standardized instrument to monitor recovery of activities and participation in children and adolescents after acute brain injuries. During their inpatient neurorehabilitation, all patients underwent one or more RemiPro assessments. For this study, we used these systematically acquired data to dichotomize outcome:

- Patients who regained the ability to interact with their environment (RemiPro: “Communication Level” or higher) during the first 22 weeks after the anoxic event were classified as “less severe outcome” (*n* = 21; marked in green (online version)/light gray (printed version) in all figures).
- Patients who, 22 weeks after the event, still showed no evidence for any interaction with their environment (RemiPro: “Perception Level” or lower) were classified as “particularly severe outcome” (*n* = 24; marked in red (online version)/dark gray (printed version) in all figures).

The cut-off of 22 weeks was chosen since, in our cohort, this was the latest time point at which a patient regained the ability to interact. Note that “interaction” in this study included all kinds of non-verbal interaction, e.g. pressing a button, or eye-closing as a “yes”-code—which implies that many patients who reached this level still showed severe disabilities—however “less severe” than the patients who did not start interacting within 22 weeks after the anoxic event. Two patients could not be classified, as their last available RemiPro assessment was performed before this 22-week cut-off and they had not yet shown any interaction by that time, leaving 45 patients with classifiable outcomes.

MRI during these first 22 weeks after the event was available for 38/45 patients with classifiable outcomes. Most of these MRI scans had been obtained before transferring the patient for neurorehabilitation, hence in many different hospitals, using different scanners and imaging protocols. We can therefore not provide any technical information other than the imaging modality (DWI/ADC, T1, T2, FLAIR). In total, 76 MRI scans (1–5 per patient) were reviewed by an experienced pediatric neurologist (M.S.), with support from a neuroradiologist (I.M.) in ambiguous cases. Both were unaware of the results of the outcome categorization. Fourteen brain regions (see Fig. 3 and 4) were assessed for abnormal signal intensities on DWI/ADC, FLAIR, T2 or T1. DWI/ADC was labeled as “abnormal” only when diffusion restriction was evident (signal hyperintensity in DWI and hypointensity in ADC). Note that we looked for restricted diffusion also beyond the typical time period of pseudonormalization in order to identify potential secondary changes as well. On FLAIR and T2, hyperintensities were noted as pathological, and hypointensities on T1.

**Fig. 2:**
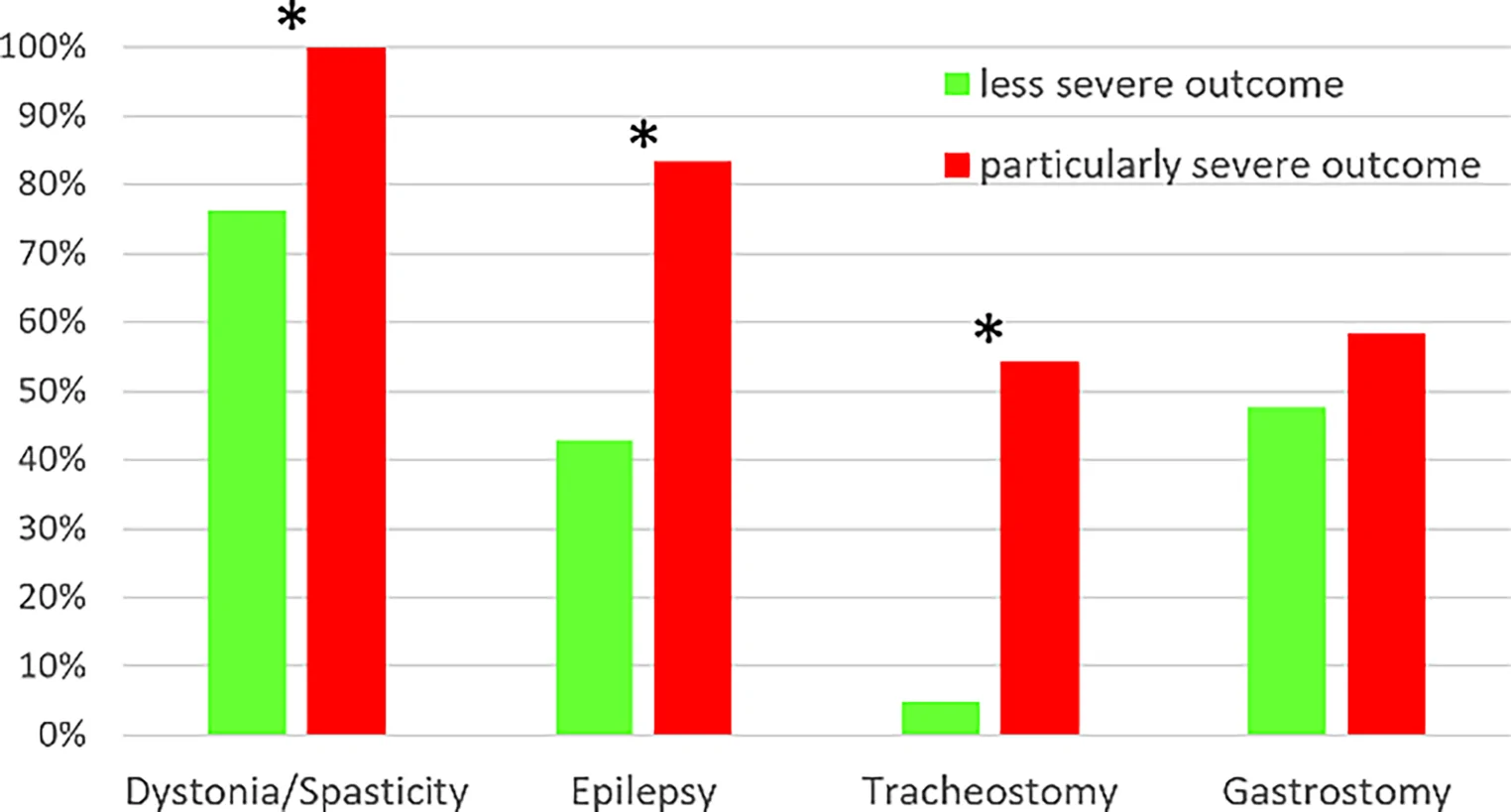
Major neurological sequelae. Prevalence (in %) of dystonia/spasticity, epilepsy, tracheostomy and gastrostomy in patients with particularly severe outcomes and less severe outcomes. Significant differences are marked with asterisks

**Fig. 3:**
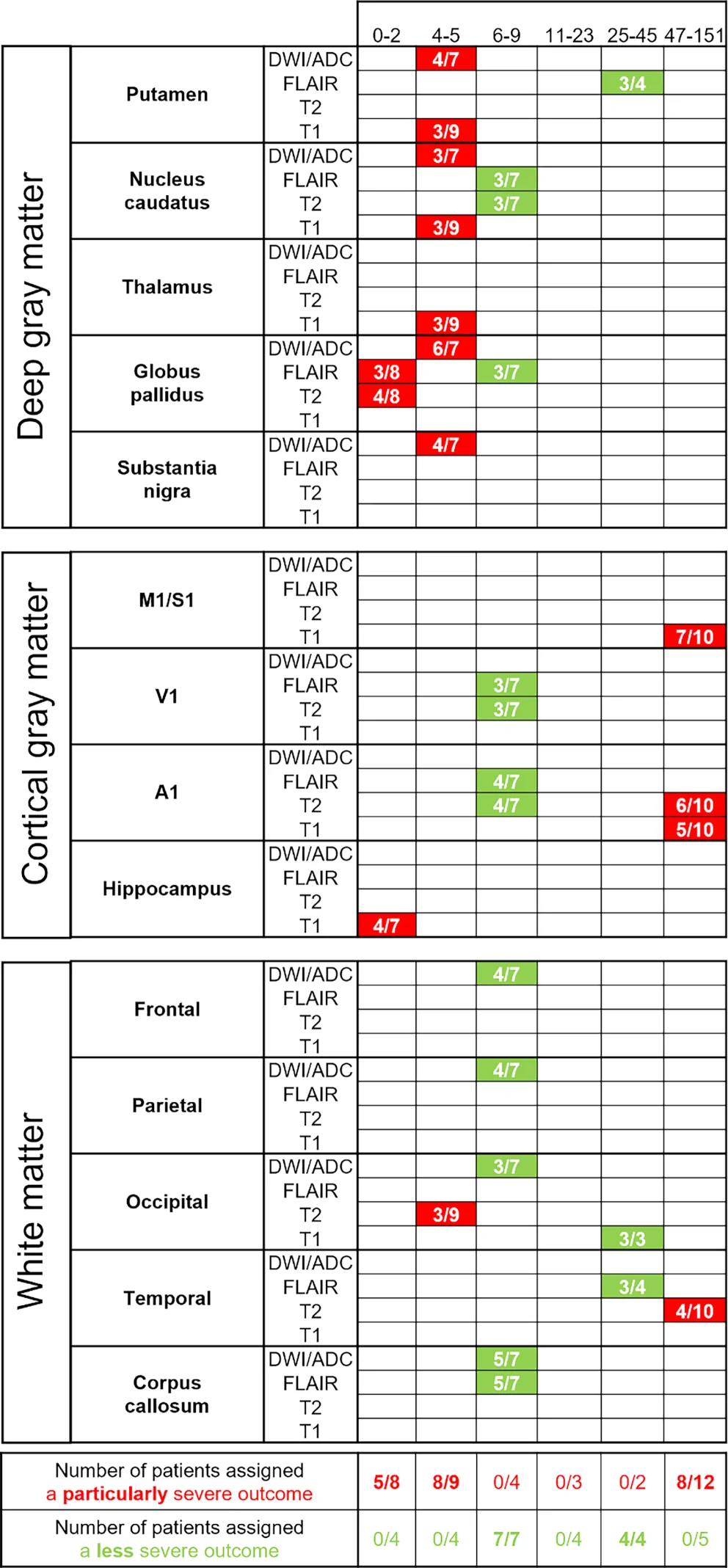
Predictive brain structures, sensitivities and specificities. *Top: *For each time interval and MRI modality (DWI/ADC, FLAIR, T2, T1), brain structures showing predictive values of 100% are marked in color (red/dark gray = signal change always predicted particularly severe outcome; green/light gray = absence of signal change always predicted less severe outcome). The white ratios (of numbers of patients) show the sensitivities (red/dark gray fields) or specificities (green/light gray fields) for the respective brain structure, modality and time interval. *Bottom:* Sensitivities and specificities for correct predictions on a patient level. Note that not all modalities were available for all MRI examinations, hence the varying denominators. M1/S1 = Primary sensorimotor cortex (pre- and postcentral gyrus); V1 = primary visual cortex (calcarine sulcus); A1 = primary auditory cortex (Heschl’s gyrus)

**Fig. 4:**
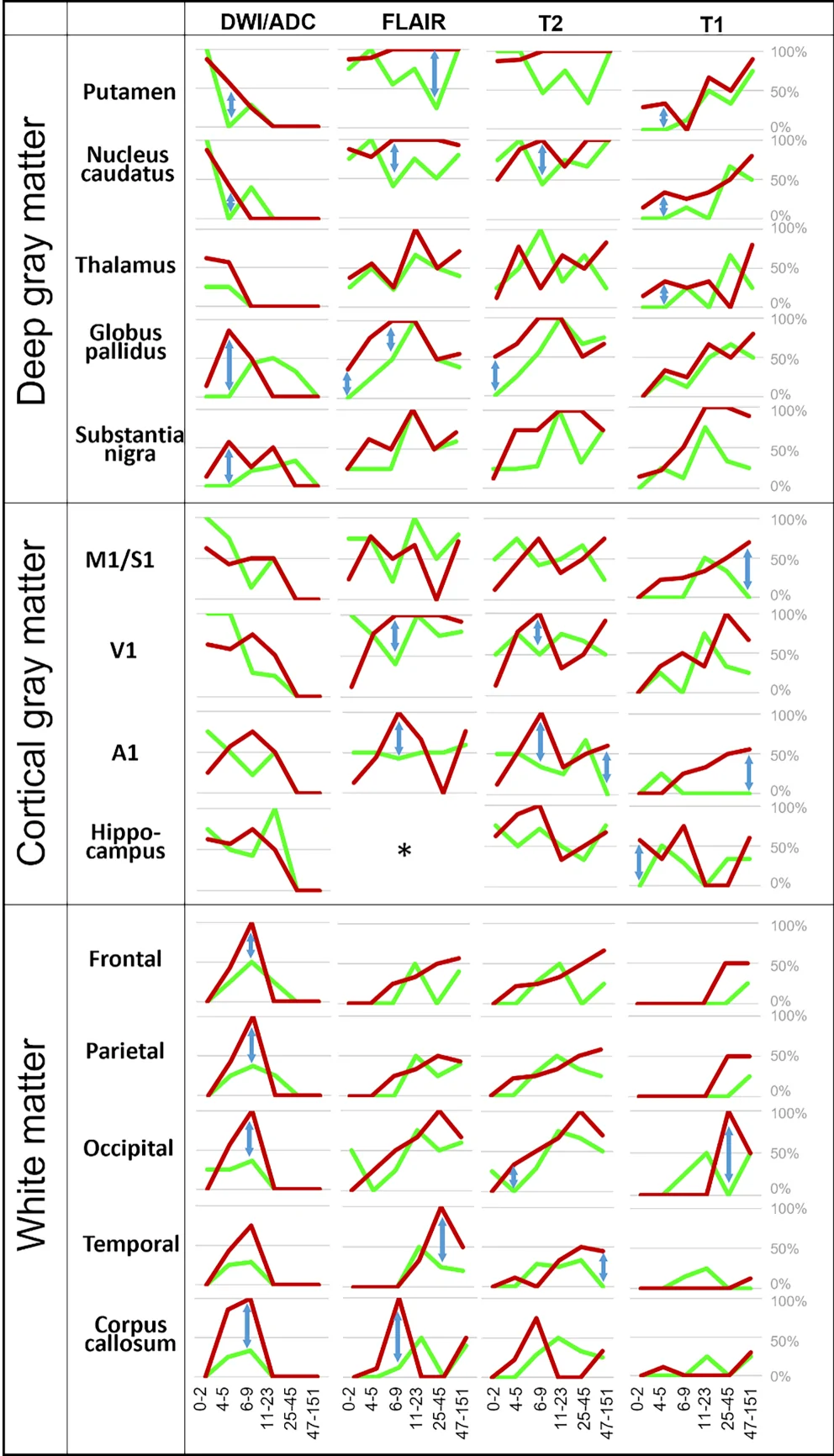
Time courses of signal abnormalities. Proportion (in percent) of patients (red/dark gray = particularly severe outcome, green/light gray = less severe outcome) with abnormal signal intensities across different time intervals, MRI modalities (DWI/ADC, FLAIR, T2, T1) and brain structures. The x‑axes represent “days after the anoxic event”. Vertical (blue) arrows indicate findings with predictive values of 100% (positive or negative) that were based on at least two patients. *FLAIR abnormalities for the hippocampus were not assessed, due to the difficulties in discriminating normal from (bilaterally) abnormal signal hyperintensities in this structure. M1/S1 = Primary sensorimotor cortex (pre- and postcentral gyrus); V1 = primary visual cortex (calcarine sulcus); A1 = primary auditory cortex (Heschl’s gyrus)

For further evaluation (see below), we grouped the MRI scans into time intervals (see Fig. 3 and 4), ensuring that each interval included at least five patients per MRI modality (DWI/ADC, FLAIR, T1 and T2). To avoid bias, we included only one MRI per patient in each time interval; when multiple MRIs were available for a patient, we selected either the technically superior or the most recent scan for analysis.

Finally, we calculated positive/negative predictive values as well as sensitivities/specificities for each combination of time interval, MRI modality and brain structure. For the presence of signal abnormality in any given brain structure, time period and MRI modality, we calculated positive predictive values as the ratios (patients with particularly severe outcomes/all patients). Likewise, for the absence of signal abnormality in any given brain structure, time period and MRI modality, we calculated negative predictive values as the ratios (patients with less severe outcomes/all patients). For further analysis, we accepted only situations with predictive values of 100%, i.e. abnormal signal occurring *only *in patients with particularly severe outcomes (“positive predictive value = 100%”) or *never* occurring in patients with particularly severe outcomes (“negative predictive value = 100%”), and in which this was based on at least two patients in order to ensure robustness. Sensitivities were calculated for the presence of signal abnormalities in any given brain structure, time period and MRI modality as the ratios (patients with particularly severe outcomes showing this signal abnormality/all patients with particularly severe outcomes). Likewise, specificities were calculated for the absence of signal abnormality in any given brain structure, time period and MRI modality as the ratios (patients with less severe outcomes not showing this signal abnormality/all patients with less severe outcomes). On a patient level, we calculated for each time period sensitivities as the ratios (patients with MRI indicating particularly severe outcome in at least one brain structure and modality/all patients with particularly severe outcomes), and specificities as the ratios (patients with MRI indicating less severe outcome in at least one brain structure/all patients with less severe outcomes).

This is a retrospective observational study. The Ethics Committee of the Eberhard-Karls-University Tübingen has confirmed that no ethical approval is required (14 Jan 2021).

## Results

As expected, given “inpatient neurorehabilitation” as an inclusion criterion, our cohort had a high prevalence of major neurological sequelae such as dystonia/spasticity or epilepsy. In addition, many patients had required tracheostomies and gastrostomies. These problems were significantly more prevalent in patients with “particularly severe outcomes” (Fig. 2).

**Fig. 5:**
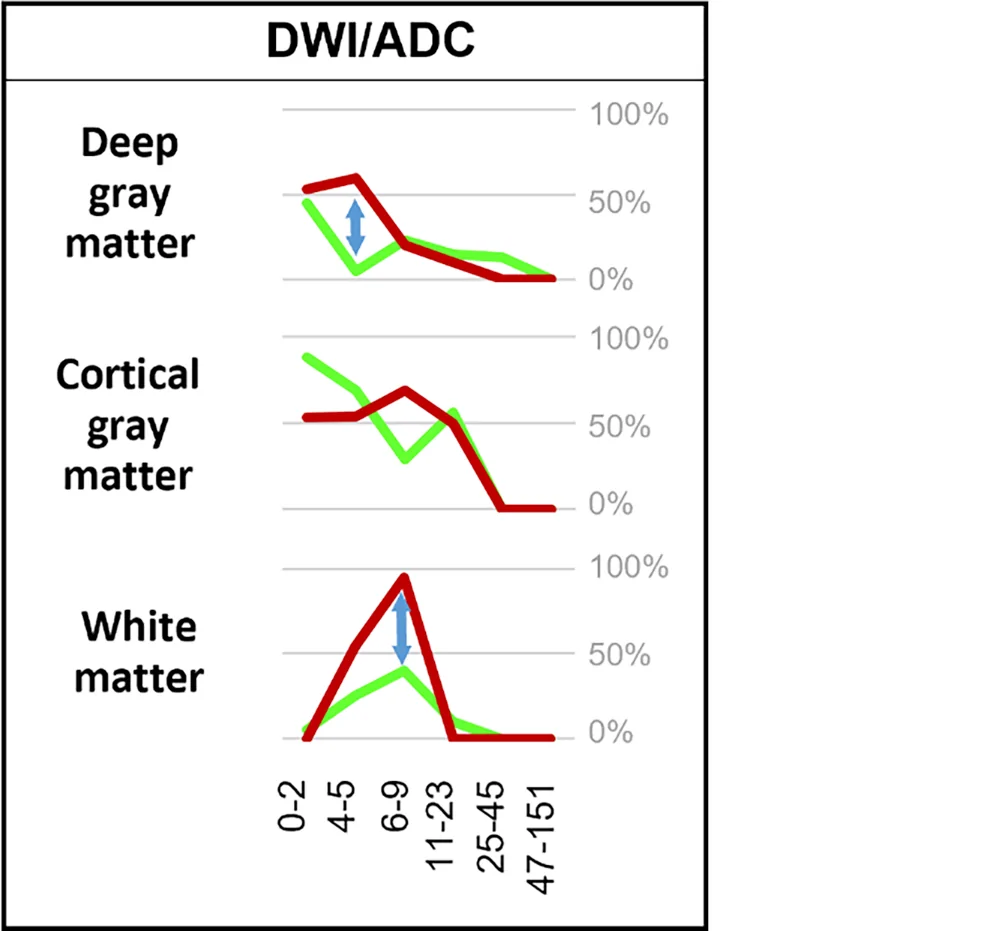
Averaged time courses of diffusion restrictions and pseudonormalization. The graphs show the mean of the proportions of patients with signal abnormalities for each tissue category (deep gray matter, cortical gray matter, white matter) and time interval after the anoxic event (as displayed in Fig. 4), separately for patients with particularly severe (red/dark gray) and less severe outcome (green/light gray). Note that diffusion restrictions beyond the 25th day were rare (only one patient in the substantia nigra on day 39, and one patient in the globus pallidus on day 45—both with less severe outcomes)

MRI signal abnormalities in deep gray matter and cortical areas were observed already during the first two days after the event, while abnormalities in the white matter started to occur only on day 4 (Fig. 4). For a better illustration of the temporal evolution of diffusion changes in the different tissue classes, we have averaged the time courses for deep gray matter, cortical gray matter and white matter in Fig. 5.

In the 14 predefined brain regions, abnormal MRI findings were more frequently observed in the patients with particularly severe outcomes (Fig. 4). Situations in which (positive or negative) predictive values of 100% were reached (and were based on data from at least two patients) are marked with arrows (blue in online version) in Fig. 4 and colored/gray fields in Fig. 3.

**Fig. 6:**
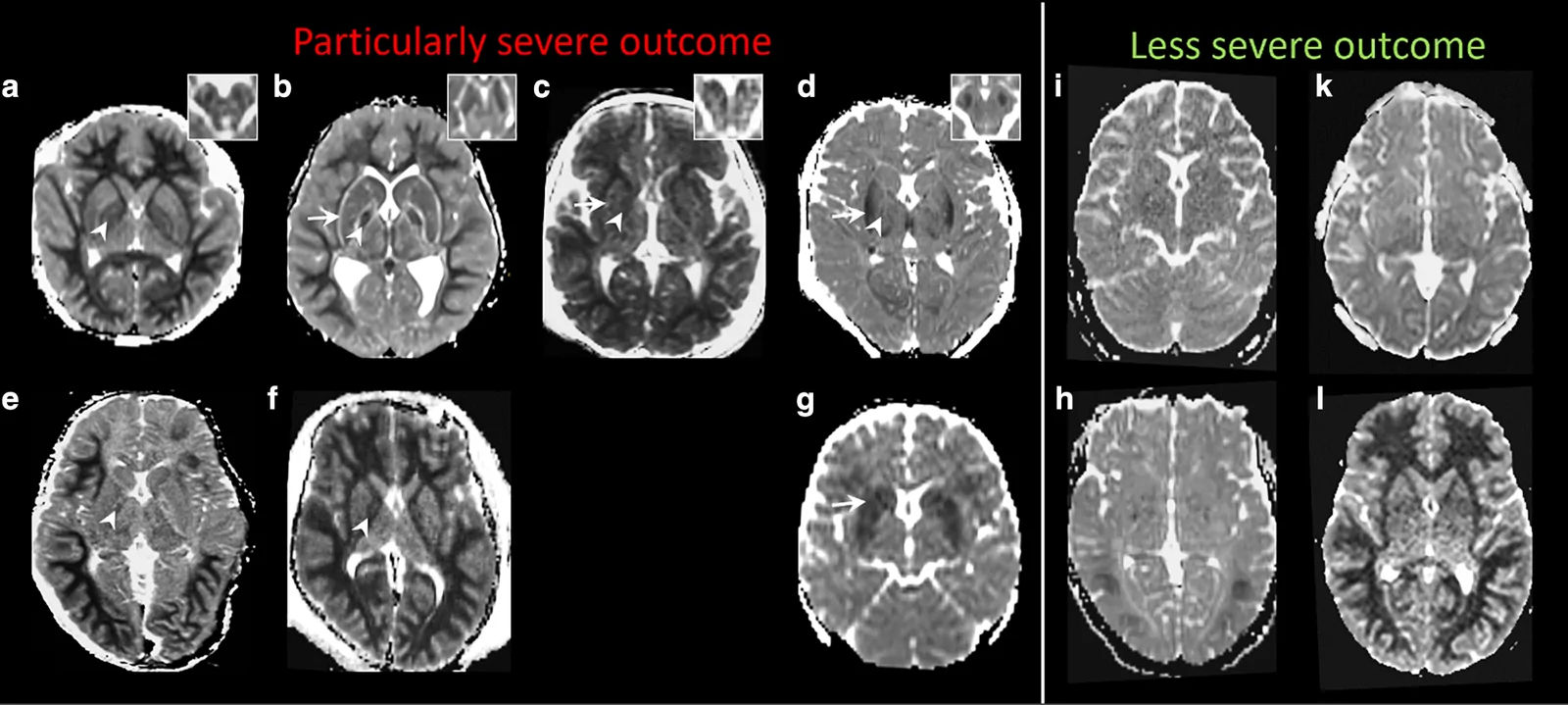
ADC-maps obtained 4–5 days after the anoxic event. All 11 patients who received DWI/ADC MRI on days 4–5 after the anoxic event are represented by one axial image each. Particularly severe outcomes (seven patients; left) occurred in all patients showing restricted diffusion (dark on ADC) in any of the following deep gray matter structures: in the globus pallidus (6/7; **a**–**f**; arrowheads), in the caudate nucleus or putamen (4/7; **b**–**d**,**g**; arrows) and in the substantia nigra (4/7; **a**–**d**; inserts). In contrast, the four patients without restricted diffusion in any of these structures (right, **h**–**l**) all had less severe outcomes. Note the diffusion restrictions in the white matter in patients (**a**–**c**,**e**,**f**) and also in patients (**h**) and (**l**): In our data, this feature was not yet predictive during this time interval. Some days later, 6–9 days after the anoxic event, however, the *absence* of such signal alterations would predict less severe outcome (cp. Figure 3 and 4)

**Fig. 7:**
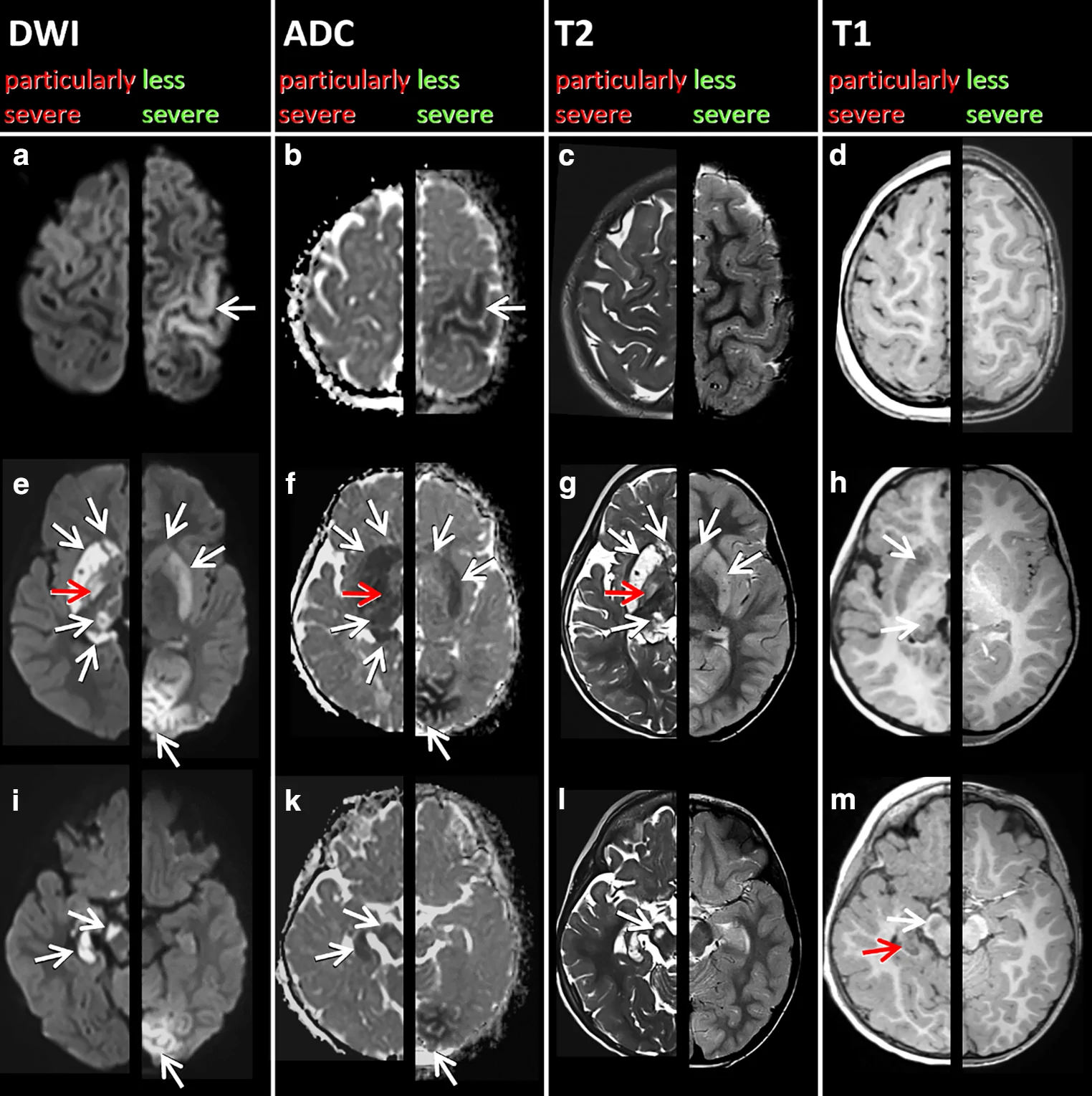
MRI obtained within 0–2 days after the anoxic event. Structures with abnormal signal intensities are marked with arrows (red/dark gray = predicting particularly severe outcome). Putamen and caudate nucleus (the “striatum”) were affected in almost all patients (arrows in **e**–**h**). Many patients showed also involvement of the thalamus (arrows in **e**–**h**), of primary cortical areas [i.e. of the primary sensorimotor cortex M1/S1 (arrows in **a**,**b**), of the primary visual cortex V1 (arrows in **e**,**f**,**i**,**k**), of the primary auditory cortex A1 (not shown)] and of the hippocampus (arrows in **e**–**m**)

**Fig. 8:**
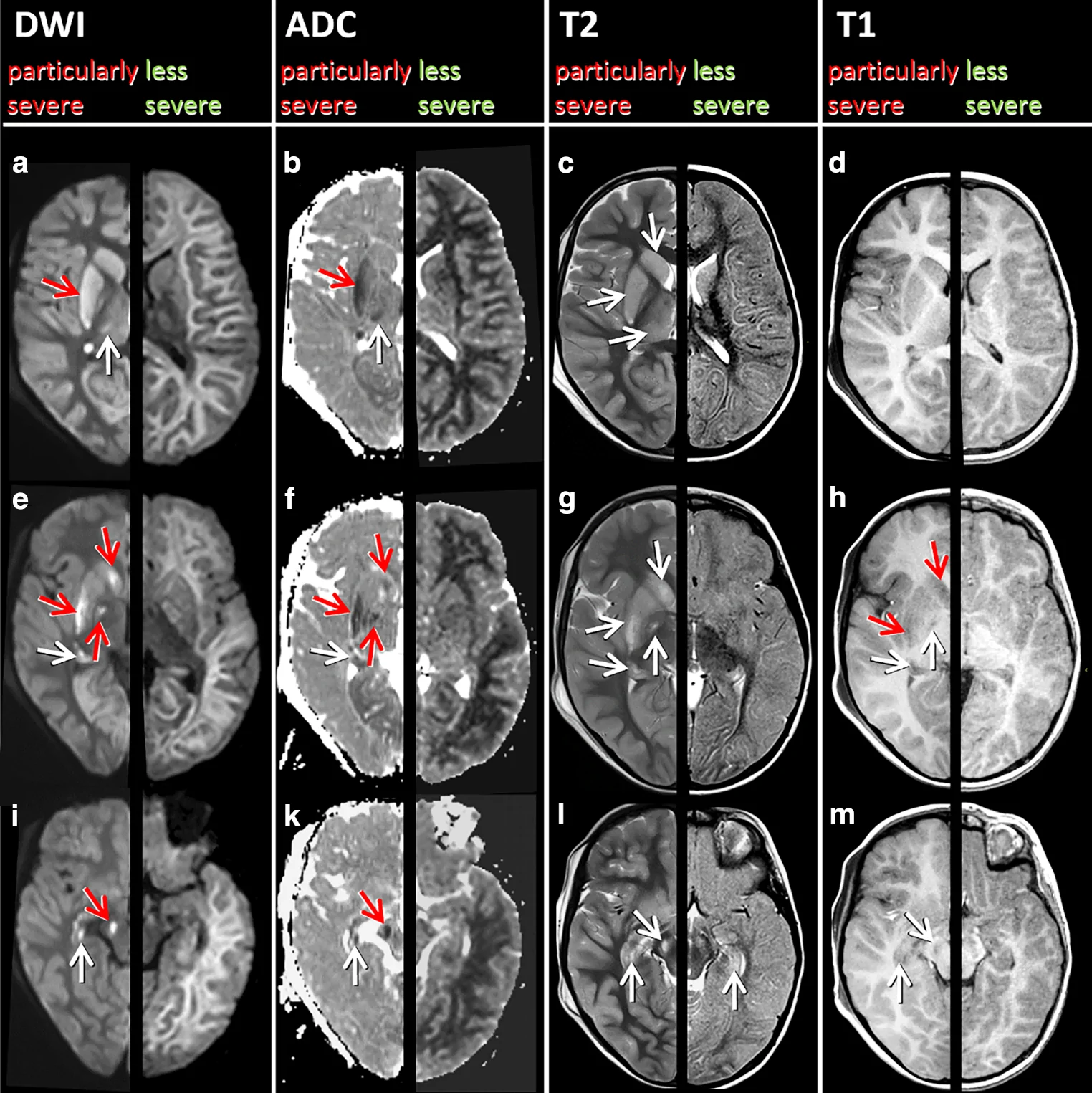
MRI obtained 4–5 days after the anoxic event. Structures with abnormal signal intensities are marked with arrows (red/dark gray = predicting particularly severe outcome). Particularly severe outcomes could be predicted by diffusion abnormalities in the globus pallidus (red/dark gray arrows in **e**,**f**), in the substantia nigra (red/dark gray arrows in **i**,**k**) and in the putamen and the caudate nucleus (red/dark gray arrows in **a**,**b**,**e**,**f**). Particularly severe outcomes could furthermore be predicted by T1-hypointensities in the putamen and the caudate nucleus (red/dark gray arrows in **h**). Note that diffusion abnormalities began to occur now also in the cerebral white matter in some patients (**a**,**b**,**e**,**f**,**i**,**k**—right sides), however this was not yet predictive. Note the—also non-predictive—characteristic appearance of cortical laminar necrosis [34] in **c**,**g**,**l**—right sides

**Fig. 9:**
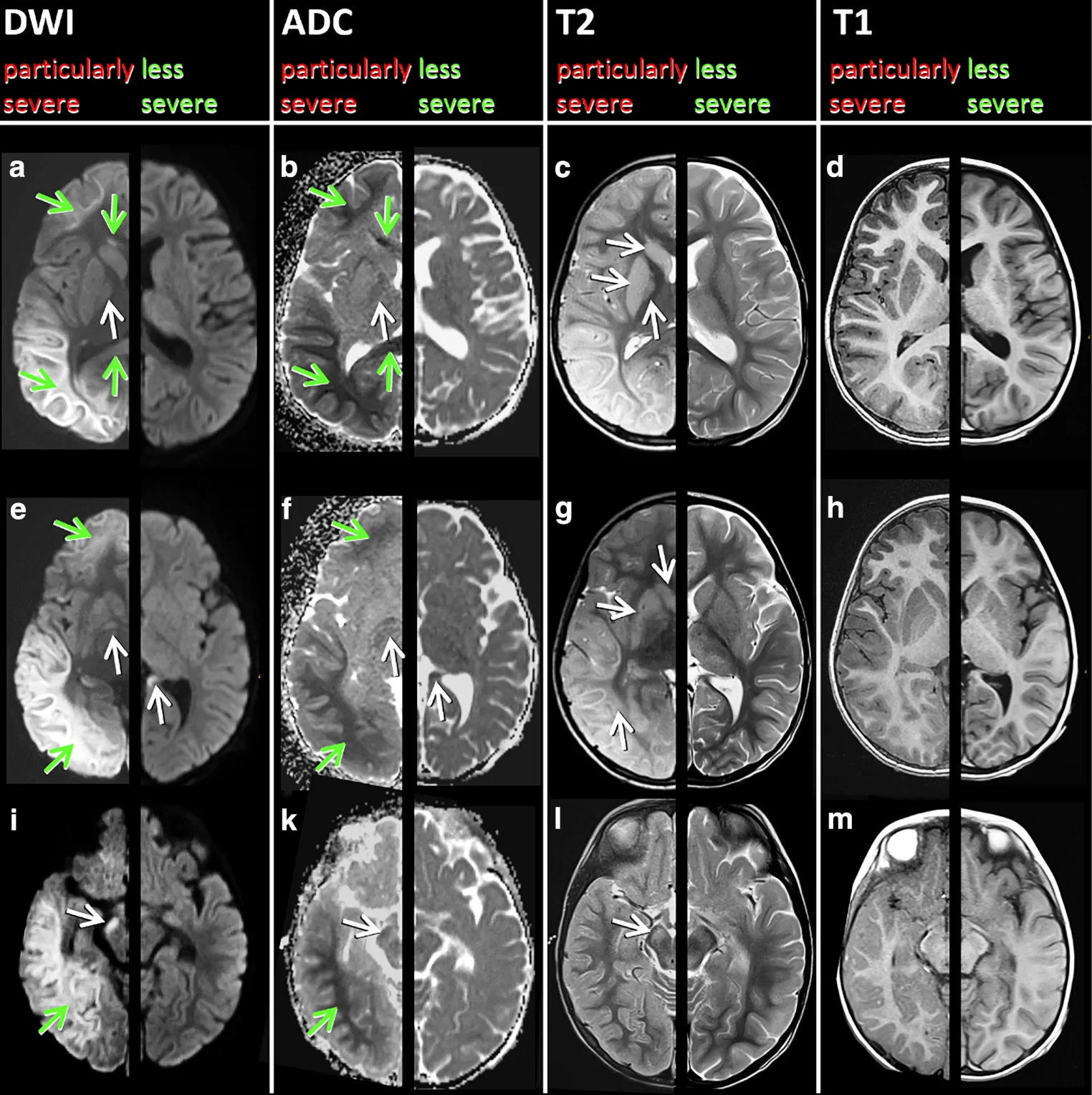
MRI obtained 6–9 days after the anoxic event. Structures with abnormal signal intensities are marked with arrows (green/light gray = absence of these signal alterations predicts less severe outcome). MRI during this time was characterized by a peak in diffusion restrictions in the cerebral white matter (Fig. 4 and 5 bottom). Now, the absence of these diffusion abnormalities in the cerebral white matter predicted less severe outcomes (green/light gray arrows)

The most informative time window for the prediction of particularly severe outcome via MRI (Fig. 3) was 4–5 days after the event, with a total of six brain structures in which signal abnormalities in one of the MRI modalities (diffusion: four structures; T1: three structures; T2: one structure) predicted particularly severe outcomes (red boxes in Fig. 3). Figure 6 displays DWI/ADC maps of all 11 patients scanned during this optimal time interval. Some predictive information could also be drawn from MRI obtained between days 0–2 and 6–9 (Fig. 3). In contrast, MRI obtained between days 11–23 no longer yielded structures with 100% predictive values (based on ≥ 2 patients), and only a few predictive structures were identified in the later time intervals (Fig. 3). Typical MRI findings acquired during the first days after the anoxic event are displayed in Fig. 7 (days 0–2), Fig. 8 (days 4–5), and Fig. 9 (days 6–9), separately for patients with particularly severe outcomes (left columns) and less severe outcomes (right columns). For each of these three figures, we chose axial images at three different levels so that a maximum of brain structures with 100% predictive values could be shown. Note that, in order to optimally display these signal changes, we used images from one patient per row of images (DWI/ADC, T2, T1), but sometimes of different patients within a figure.

## Discussion

The major finding of our study was that MRI can indeed identify patients likely to develop particularly severe outcomes already within the first few days following an acute anoxic event. This suggests that MRI has the potential to provide valuable prognostic information during the early intensive care phase—when decisions about the continuation or withdrawal of life-sustaining therapies may be under consideration.

Based on our findings, we recommend obtaining MRI for such prognostic purposes no later than day 9, with the optimal time window between days 4 and 5 after the event. During this time period, we would have correctly predicted particularly severe outcomes in 8/9 patients (Fig. 3, bottom). Furthermore, for MRI obtained during this period, we could identify the highest number of different brain structures (six) carrying predictive information in one or two MRI modalities (Fig. 3, top). This recommendation aligns broadly with current recommendations published by the American Heart Association (days 3–7) [15] and the International Liaison Committee on Resuscitation (days 3–14) [16]. Notably, our data did not include any patients scanned exactly on day 3, which prevents us from evaluating the predictive value of this specific time point included in both recommendations [15, 16].

All MRI modalities we analyzed in this study (DWI/ADC, T1, T2, FLAIR) carried valuable information (see colored boxes in Fig. 3) and should therefore be included in MRI protocols for such situations. As expected, diffusion MRI was the most valuable modality during the first days after the anoxic event (in fact, no diffusion images were available in the one patient in whom we would have missed the prediction of particularly severe outcome despite MRI obtained between 4–5 days after the event). T1, T2 and FLAIR become more important for the—more difficult—predictions beyond the first two weeks following the anoxic event.

In addition to its prognostic implications, our study contributes to understanding the temporal evolution of MRI signal abnormalities, and especially of DWI changes, following acute anoxia. For illustration of this evolution, we have averaged the time courses for DWI changes in the structures of deep gray matter, cortical gray matter and white matter (Fig. 5). We observed early diffusion restrictions in deep and cortical gray matter (starting at days 0–2), but delayed development of diffusion restrictions in the white matter (starting only at days 4–5, peaking at days 6–9). To our knowledge, these time courses have not been reported before in comparable detail. Previous reports on MRI after anoxic events in childhood and adolescence fit, however, well into this scenario [4–7, 17–23].

From a pathophysiological perspective, early gray matter signal changes are widely interpreted as reflecting “cytotoxic edema” [5, 6]. In contrast, variable explanations have been put forward for the delayed white matter changes developing only several days after the event. With the relatively large number of patients showing this phenomenon in our study (16 patients in total), we were able to delineate the time course for the appearance and disappearance of this sign (Fig. 5 bottom; Fig. 9a, b—left). We propose that these changes reflect secondary axonal damage, similar to Wallerian degeneration [24, 25] (or indeed preceding Wallerian degeneration, hence the expression “pre-Wallerian degeneration” used in [26, 27]), following the primary anoxic damage to neurons in cortical and deep gray matter. A similar phenomenon has recently been reported after surgical disconnection of white matter tracts [28]. In our cohort, we could demonstrate the prognostic value of this phenomenon: When MRI obtained on days 6–9 did *not* show diffusion restrictions in the cerebral white matter, the outcome was always less severe. Note that the presence of such diffusion restrictions, although sometimes quite impressive (e.g. patient L in Fig. 6) does not necessarily predict particularly severe outcome.

The time courses displayed in Fig. 5 also depict the phenomenon of “pseudonormalization” both in gray and white matter. This term refers to the return of restricted diffusion patterns on DWI to a more normal appearance within a specific timeframe, often within 7–8 days after a hypoxic/ischemic event, despite ongoing tissue damage [29–31]. Initially, restricted diffusion, indicative of cytotoxic edema, is observed due to impaired cell membrane function trapping water molecules. Over time, the affected areas may appear similar to surrounding tissue due to a shift from cytotoxic to vasogenic edema, where fluid leaks into the extracellular space, increasing overall diffusion and ADC values [32]. In our data, pseudonormalization sometimes started before day 4 (deep gray matter & less severe outcome; Fig. 5 top), but could continue until three weeks after the anoxic event. It is tempting to speculate that, when this “delayed pseudonormalization” occurs in cortex or deep gray matter (Fig. 5), similar explanations like those proposed for the white matter (i.e., secondary axonal damage) could apply.

In deep gray matter, it was the *delay* of pseudonormalization (Fig. 5 top, blue arrow) that turned out to be the most important predictor of particularly severe outcomes: When scanned on days 0–2, almost all patients (irrespective of outcome) showed restricted diffusion in the putamen and the caudate nucleus (Fig. 4 top left; Fig. 7e, f), but only in the patients with less severe outcome, this apparently pseudonormalized until day 4 (Fig. 4 top left; Fig. 8a, b, e, f). It was therefore the *persistence* of diffusion restrictions beyond day 3 in these structures that indicated particularly severe outcomes. This observation is compatible with findings reporting worse outcomes after neonatal asphyxia when a second MRI obtained during the second week after the event still showed diffusion restriction (the authors called this “pseudonormalization negativity” [31]).

In the cortical gray matter, we observed that pseudonormalization took much longer (until day 23), without any predictive differences between patients with particularly and less severe outcomes. This temporal evolution is compatible with reports from adults with ischemic stroke, demonstrating that diffusion restrictions in the cortex only disappeared between 2 and 4 weeks after the event [33].

In the cerebral white matter of our patients, pseudonormalization was completed by day 11 in almost all patients (Fig. 5 bottom). This timing fits well with the “pseudonormalization times” of white matter structures reported in [31] after neonatal asphyxia, ranging from 8.3 and 11.5 days.

Admittedly, our study has several limitations: First, although this is the largest study so far on qualitative MRI after anoxic events in childhood and adolescence, the number of patients in each time interval remains relatively small. This may have led to effects like the predominance of “positive predictions” on days 4–5 (since 8/13 MRIs available for this time period were of patients with “particularly severe outcomes”) contrasting with “negative predictions” on days 6–9 (a time period in which 7/11 MRIs were of patients with “less severe outcomes”), or the absence of predictive structures on days 11–23. All results must therefore be interpreted with caution, and larger studies are needed to confirm and expand upon these results. The small sample size was also the reason why we could not split our cohort into different age ranges, e.g. comparing toddlers with adolescents. Second, we used RemiPro for dichotomizing neurological outcome. This instrument was originally designed for neurorehabilitation, and not specifically designed to guide decisions concerning the continuation or withdrawal of life-supporting therapies. Therefore, our study should be regarded as a “proof-of-principle”, demonstrating that early MRI has the potential to identify patients with particularly severe outcomes. On the other hand, we are not aware of any instrument that has been developed or validated for this purpose, highlighting the urgent need for such an instrument. Third, this retrospective study completely relies on data generated during routine clinical care in many different institutions. Therefore, we cannot exclude selection bias, e.g. in the decisions when and for which patients MRI had been performed in the acute phase. Fourth, although no patient in our cohort died during neurorehabilitation before the 22-week cut-off, we have no information on patients who died before transfer to neurorehabilitation—with or without withdrawal of life-sustaining therapies.

In summary, we could demonstrate that early MRI has the potential to identify children and adolescents at risk of particularly severe outcomes after acute anoxic events. Our data suggest obtaining MRI for prognostic purposes ideally on days 4–5 after the event, when persisting DWI/ADC abnormalities in several structures of the deep gray matter (putamen, caudate nucleus, globus pallidus, substantia nigra) consistently indicated particularly severe outcomes. Larger and more systematic studies, particularly those incorporating outcome measures tailored to critical care decision-making, are urgently needed.

## Data Availability

No datasets were generated or analysed during the current study.
